# The burden of diabetes mellitus during pregnancy in low- and middle-income countries: a systematic review

**DOI:** 10.3402/gha.v7.23987

**Published:** 2014-07-01

**Authors:** Lovney Kanguru, Navya Bezawada, Julia Hussein, Jacqueline Bell

**Affiliations:** 1 IMMPACT, School of Medicine & Dentistry, University of Aberdeen, Scotland, UK; 2 Medical School, School of Medicine & Dentistry, University of Aberdeen, Scotland, UK

**Keywords:** diabetes mellitus, gestational diabetes mellitus, non-communicable diseases, pregnancy, prevalence

## Abstract

**Background:**

Little is known about the burden of diabetes mellitus (DM) in pregnancy in low- and middle-income countries despite high prevalence and mortality rates being observed in these countries.

**Objective:**

To investigate the prevalence and geographical patterns of DM in pregnancy up to 1 year post-delivery in low- and middle-income countries.

**Search strategy:**

Medline, Embase, Cochrane (Central), Cinahl and CAB databases were searched with no date restrictions.

**Selection criteria:**

Articles assessing the prevalence of gestational diabetes mellitus (GDM), and types 1 and 2 DM were sought.

**Data collection and analysis:**

Articles were independently screened by at least two reviewers. Forest plots were used to present prevalence rates and linear trends calculated by linear regression where appropriate.

**Main results:**

A total of 45 articles were included. The prevalence of GDM varied. Diagnosis was made by the American Diabetes Association criteria (1.50–15.5%), the Australian Diabetes in Pregnancy Society criteria (20.8%), the Diabetes in Pregnancy Study Group India criteria (13.4%), the European Association for the Study of Diabetes criteria (1.6%), the International Association of Diabetes and Pregnancy Study Groups criteria (8.9–20.4%), the National Diabetes Data Group criteria (0.56–6.30%) and the World Health Organization criteria (0.4–24.3%). Vietnam, India and Cuba had the highest prevalence rates. Types 1 and 2 DM were less often reported. Reports of maternal mortality due to DM were not found. No geographical patterns of the prevalence of GDM could be confirmed but data from Africa is particularly limited.

**Conclusion:**

Existing published data are insufficient to build a clear picture of the burden and distribution of DM in pregnancy in low- and middle-income countries. Consensus on a common diagnostic criterion for GDM is needed. Type 1 and 2 DM in pregnancy and postpartum DM are other neglected areas.

Diabetes mellitus (DM) is a metabolic disorder resulting from a defect in insulin production, impaired insulin action or both. It is one of the major non-communicable diseases on the rise worldwide, causing 4.8 million deaths and morbidity in 371 million people every year (1). In recent years, patterns of change have been observed in the age of onset of DM with younger populations now disproportionately affected. It is currently estimated that 28 million women of reproductive age suffer from DM worldwide (2). Majority of these women have type 2 DM, and 80% of the burden is found in low- and middle-income countries (2).

In pregnancy, DM can either be pre-existing (type 1 or 2) or gestational diabetes mellitus (GDM). In pre-existing DM, risk factors such as genetic predisposition, family history of type 1 DM and autoimmune disorders are crucial in the development of type 1 DM (3, 4). Factors which play a significant role in both type 2 DM and GDM include obesity, unhealthy diets, physical inactivity, family histories of type 2 DM, maternal age and ethnicity (4, 5). Other lifestyle changes such as alcohol abuse and smoking have also been implicated in the aetiology of type 2 DM (6).

A diabetic pregnant woman and her unborn child are at increased risk of pregnancy complications such as pre-eclampsia, infections, obstructed labour, postpartum haemorrhage, preterm births, stillbirths, macrosomia, miscarriage, intrauterine growth retardation, congenital anomalies, birth injuries and death in worst case scenarios (7, 8). Women are also at risk of long-term diabetic complications, including retinopathy, nephropathy and neuropathy.

Beyond the 42-day postpartum period, consequent effects of DM in pregnancy can also be seen. An estimated 30–50% of women with a previous history of GDM develop it again in subsequent pregnancies, and within 5–10 years, 50% of these women will develop type 2 DM (9–11). In addition, babies born from diabetic pregnancies have an increased risk of developing obesity in childhood, metabolic disturbances in adolescence and type 2 DM in adulthood, linked to the metabolic imbalance experienced in utero (3).

Appropriate diagnosis, care and management of DM in the pre-pregnancy, pregnancy and post-pregnancy periods are important to minimise the risk of complications, long-term effects or catastrophic death of the mother and/or baby (12). Several diagnosing criteria for GDM are used worldwide. These include the ADA (America Diabetes Association), ADIPS (Australian Diabetes in Pregnancy Society), DIPSI (Diabetes in Pregnancy Study Group India), EASD (European Association for the Study of Diabetes), IADPSG (International Association of Diabetes and Pregnancy Study Groups), NICE (National Institute of Health and Clinical Excellence), NDDG (National Diabetes Data Group), SIGN (Scottish International Guidelines Network) and WHO (World Health Organization for both pregnant and non-pregnant populations) (13, 14). These criteria differ in the group screened (universal or only high-risk women), gestational age at screening, loading dose for the oral glucose tolerance test (OGTT) and the OGTT cut-off levels of plasma glucose.

In some of the poorest areas of the world, difficulties in accessing and receiving both maternity and general medical care increase the risks pregnant women face from the complications of diabetes in pregnancy. It is estimated that women with type 1 DM face a 5–20% risk of dying in pregnancy compared to non-diabetic pregnant women if adequate care is not provided (15).

Despite the high burden of diabetes in low- and middle-income countries, little is known about the contribution of DM in pregnancy in these countries. This review aims to investigate the prevalence and geographical pattern of DM (pre-existing and gestational) in pregnancy and up to 1 year post-delivery in low- and middle-income countries. We took 1 year as the cut-off point because it is up to this period that late maternal deaths are recorded (worldwide) and is also jointly agreed by the WHO, UNFPA, UNICEF and the World Bank (16).

## Methods

A priori protocol was written before undertaking the review and the PRISMA statement used to guide reporting (17).

### Inclusion and exclusion criteria

Randomised, non-randomised and observational study designs of primary or secondary studies were eligible for inclusion if they reported on prevalence and/or mortality rates due to any type of DM in pregnancy up to 1 year after childbirth. Editorials, letters, commentaries and short notes were excluded. Systematic reviews were not eligible for inclusion; however, their references were screened for relevant primary or secondary studies. We also excluded studies that had modelled or extrapolated prevalence or mortality estimates.

Studies that looked at pregnant women with pre-existing DM (type 1 and 2) or GDM confirmed by any international diagnostic criteria, for example, the ADA, ADIPS, DIPSI, EASD, IADPSG, NDDG, NICE, SIGN and WHO, were included. Studies with women up to 1 year since their last delivery with confirmed diagnosis of diabetes were also eligible for inclusion. Studies regarding non-diabetic pregnant women, diabetic women who had delivered more than 1 year ago and self-reported diabetic women with no clinical and diagnostic confirmatory tests were excluded.

Prevalence of GDM and DM (type 1 and 2) in pregnancy up to 1 year post-delivery was the primary outcome measure. Mortality due to GDM and DM (type 1 and 2) in pregnancy up to 1 year post-delivery was the secondary outcome measure; screening criteria, gestational age, parity, maternal age and setting were included as explanatory outcome measures. Prevalence or mortality related to impaired glucose tolerance (IGT) and metabolic syndrome were excluded. All studies which were carried out in countries listed by the World Bank as low, lower and upper middle-income countries were considered for inclusion (18).

### Electronic searches

A comprehensive search of Medline, Medline-in-process, Embase, CAB abstracts, Cochrane Central Register of Controlled Trials and Cinahl databases was conducted using appropriate MeSH terms combined by Boolean commands ‘AND’ and ‘OR’. Key words in the search strategy included (diabetes OR type 1 diabetes OR juvenile diabetes OR child diabetes OR autoimmune diabetes OR insulin-dependent diabetes OR DM OR type 2 diabetes OR adult onset diabetes OR non-insulin-dependent diabetes OR gestational diabetes) AND (maternal mortality OR maternal morbidity OR pregnancy OR pregnant women OR pregnancy complications) AND (developing countries OR low-income countries OR lower income countries OR low- and middle-income countries OR upper middle-income countries). Reference lists of included studies and review papers were screened for relevance and hand searching of relevant reports done. Although the Cochrane collaborative strongly advises against setting language restrictions to prevent effects of possible language bias by exclusion of articles (and study populations) published in non-English journals, articles in the English language were the only ones eligible for inclusion due to financial constraints tied to translation costs of the non-English papers. There were no date restrictions and all the searches ran until March 2014.

### Data management and extraction

Reference Manager (version 12) was used to manage all of the citations retrieved. Two reviewers (LK, NB) initially screened titles and abstracts independently using the inclusion–exclusion criteria. Relevant articles were selected and their full texts sought. These were then screened for eligibility by all of the reviewers (LK, NB, JH and JB) independently, ensuring at least two reviewers screened each article. Where disagreements arose about inclusion of an article, discussions resolved these. A data extraction form was developed incorporating important characteristics such as study design, country, sampling frame, sample size and relevant outcomes.

## Data synthesis and analysis

Table 1 and 2 were used to summarise characteristics of the included studies: one showing a general methodological description of the studies and the second showing outcome measures of interest. Prevalence of GDM and type 1 and 2 DM were computed. 95% confidence intervals of GDM prevalence were calculated and compiled (Fig. 2) using metadata viewer for epidemiological studies (version 1, March 2011). The overall prevalence results could not be pooled together by a meta-analysis due to underlying clinical heterogeneity such as differences in the gestational age for screening, maternal age and different criteria used which were all likely to influence the results and also lack of a comparator group for most studies. As part of the exploration of geographical patterns of prevalence, the (rural or urban) setting of the study was identified. Association between GDM and gross national income (GNI) per capita (19) was determined using a linear regression model and a scatter plot was used to illustrate findings (Fig. 3).

**Table 1:** Description of included studies

Author, year	Country	Study design	Setting	Sampling frame	Sample size	
1. Akter et al. (1996)	Pakistan	Retrospective cohort	Unclear	Tertiary hospital	6,830	
2. Jawad et al. (1996)	Pakistan	Prospective cohort	Unclear	Tertiary hospital	5,559	
3. Khan et al. (1991)	Pakistan	Prospective cohort	Unclear	Tertiary hospital	1,267	
4. Ramachandran et al. (1994)	India	Cross sectional	Unclear	2 gynaecology centres	950	
5. Ramachandran et al. (1998)	India	Prospective cohort	Urban	2prenatal clinics	1,036	
6. Grewal et al. (2012)	India	Prospective cohort	Urban	Tertiary hospital	298	
7. Hill et al. (2005)	India	Prospective cohort	Urban	Tertiary hospital	785	
8. Swami et al. (2008)	India	Prospective cohort	Unclear	Secondary/ tertiary hospital	1,225	
9. Seshiah et al. (2012)	India	Prospective cohort	Unclear	Community health centres	1,463	
10. Seshiah et al. (2004)	India	Prospective cohort	Unclear	Tertiary hospital	1,251	
11. Tripathi et al. (2012)	India	Prospective cohort	Urban	Tertiary hospital	687	
12. Wahi et al. (2011)	India	Prospective cohort	Unclear	Tertiary hospital	2,025	
13. Siribaddana et al. (1998)	Sri Lanka	Prospective cohort	Unclear	Secondary/ Tertiary hospital	721	
14. Boriboonhirunsarn et al. (2004)	Thailand	Cross sectional	Urban	Tertiary hospital	1,200	
15. Chanprapaph et al (2004)	Thailand	Retrospective cohort	Urban	Tertiary hospital	1,000	
16. Lueprasitsakul et al. (2008)	Thailand	Retrospective cohort	Urban	Tertiary hospital	637	
17. Serirat, S. et al. (1991)	Thailand	Prospective cohort	Urban	Secondary/ tertiary hospital	25,997	Facility-based studies
18. Sumeksri et al. (2006)	Thailand	Prospective cohort	Urban	Secondary/ Tertiary hospital	1,332	
19. Fan et al. (2006)	China	Prospective cohort	Urban	Tertiary hospital	20,512	
20. Tran et al. (2013)	Vietnam	Prospective cohort	Urban	Tertiary hospital	2,772	
21. Hirst et al. (2012)	Vietnam	Prospective cohort	Urban	Tertiary hospitals	2,702	
22. Baci et al. (2013)	Turkey	Prospective cohort	Urban	Tertiary hospital	614	
23. Karcaaltincaba et al. (2009)	Turkey	Retrospective cohort	Urban	Tertiary hospital	21,531	
24. Kosus et al. (2012)	Turkey	Retrospective cohort	Urban	Tertiary hospital	808	
25. Erem et al. (2002)	Turkey	Cross sectional	Unclear	7 health stations	807	
26. Tanir et al. (2005)	Turkey	Retrospective cohort	Urban	Tertiary hospital	3,548	
27. Hadaegh et al. (2005)	Iran	Prospective cohort	Unclear	Obstetric clinics in Bandar Abbas city	800	
28. Hossein-Nezhad et al. (2006)	Iran	Cross sectional	Urban	5 teaching hospitals	2,416	
29. Keshavarz et al. (2005)	Iran	Prospective cohort	Urban	Tertiary hospital	1,310	
30. Ranchod, H.A. et al. (1991)	South Africa	Prospective cohort	Urban	Tertiary hospital	1,721	
31. Anzaku and Musa (2013)	Nigeria	Cross sectional	Urban	Tertiary hospital	253	
32. Olarinoye et al. (2004)	Nigeria	Prospective cohort	Urban	Tertiary hospital	293	
33. Ozumba et al. (2004)	Nigeria	Retrospective cohort	Urban	Tertiary hospital	12,030	
34. Balaji et al. (2012)	India	Cross sectional	Urban, suburban & rural	Community health centres	819	
35. Seshiah et al. (2008), (2009)	India	Cross sectional	Urban, suburban & rural	20 health posts, 10 primary & community centres	12,056	
36. Zargar et al. (2004)	India	Prospective cohort	Urban & rural	All ANC in 6 districts of Kashmiri valley	2,000	
37. Dahanayaka et al. (2012)	Sri Lanka	Cross sectional	Unclear	Three MOH areas in a district	405	
38. Sayeed et al. (2005)	Bangladesh	Prospective cohort	Rural	10 villages with union council & local government	172	
39. Yang et al. (2009)	China	Prospective cohort	Unclear	26 hospitals in 18 cities	16,286	Population-based studies
40. Yang et al. (2002)	China	Prospective cohort	Urban	All ANC units in the 6 central districts	9,471	
41. Zhang et al. (2011)	China	Prospective cohort	Urban	All secondary/ tertiary hospitals in the 6 central districts	105,473	
42. Seyoum et al. (1999)	Ethiopia	Cross sectional	Rural	18 villages in East Tigray	890	
43. McCarthy et al. (2010)	Argentina	Prospective cohort	Urban	All the 23 primary health centres in La Plata city	1,702	
44. Schmidt et al. (2000)	Brazil	Prospective cohort	Urban	All ANC in the NHS in 6 state capitals	5,004	
45. Davilla et al. (2011)	Cuba	Retrospective cohort	Unclear	All of Isle of Youth	1,003	

MOH, ministry of health; ANC, antenatal clinics; NHS, National Health Service.

**Table 2:** Characteristics of included studies (gestational diabetes mellitus, type 1 and 2 diabetes mellitus, postpartum type 2 diabetes mellitus)

Author, year	Country	Maternal age (years)	Parity (GDM only)	Gestational age at diagnosis	Diagnosing criteria	Screening criteria (GDM only)	GDM prevalence (%)	Total DM prevalence (T1, T2)	Prevalence of postpartum type 2 DM	
1. Akter et al. (1996)	Pakistan	Mean 26.9	Null=19% >1=81%	Unclear	WHO	Selective	3.30	0.6%	Not reported	
2. Jawad et al. (1996)	Pakistan	20–45	Null=21% >1=79%	Unclear	NDDG	Universal	3.45	Not reported	Not reported	
3. Khan et al. (1991)	Pakistan	22–34	Not reported	<28 weeks & 28–32 weeks	NDDG	Universal	3.20	Not reported	Not reported	
4. Ramachandran et al. (1994)	India	<20–35+	Not reported	Unclear	NDDG	Universal	0.56	0.6%	Not reported	
5. Ramachandran et al. (1998)	India	Unclear	Not reported	24–28 weeks	NDDG	Universal	0.86	0.29%	Not reported	
6. Grewal et al. (2012)	India	18–39	Not reported	1st trimester, & 24–28 weeks	ADA	Universal	15.49	Not reported	Not reported	
7. Hill et al. (2005)	India	16–40	Not reported	28–32 weeks	ADA	Universal	5.80	Not reported	Not reported	
8. Swami et al. (2008)	India	18–40	Not reported	NR	ADA	Universal	7.70	Not reported	Not reported	
9. Seshiah et al. (2012)	India	Mean 23.6 (±3.32)	Not reported	~22–34 weeks	DIPSIIADPSG	Universal	13.414.6	Not reported	Not reported	
10. Seshiah et al. (2004)	India	19–27	Not reported	Unclear	WHO	Universal	17.70	Not reported	Not reported	
11. Tripathi et al. (2012)	India	20–32	Unclear	24–28 weeks	ADA	Universal	1.50	Not reported	Not reported	
12. Wahi et al. (2011)	India	22–30	Not reported	24–28 weeks	WHO	Selective	6.94	Not reported	Not reported	
13. Siribaddana et al. (1998)	Sri Lanka	15–44	Not reported	24–28 weeks	WHO	Universal	5.50	Not reported	Not reported	
14. Boriboonhirunsarn et al. (2004)	Thailand	25–36	Unclear	7.6–16.6 weeks	NDDG	Selective	5.10	Not reported	Not reported	
15. Chanprapaph et al. (2004)	Thailand	Mean 21–33	Unclear	Unclear	NDDG	Selective	2.90	0.2%	Not reported	
16. Lueprasitsakul et al. (2008)	Thailand	Unclear	Not reported	Unclear	NDDG	Selective	1.50	Not reported	Not reported	Facility-based
17. Serirat et al. (1991)	Thailand	15–41	Null=43% >1=57%	Unclear	NDDG	Universal	2.02	Not reported	Not reported	studies
18. Sumeksri et al. (2006)	Thailand	30–34	Not reported	25.6–28 weeks	NDDG	Universal	2.40	Not reported	Not reported	
19. Fan et al. (2006)	China	26–35	Not reported	Unclear	NDDG	Universal	3.80	Not reported	Not reported	
20. Tran et al. (2013)	Vietnam	16–44	Null=36% ≥1=64%	24–32 weeks	ADA IADPSG ADIPS WHO	Selective	5.9 20.4 20.8 24.3	Not reported	Not reported	
21. Hirst et al. (2012)	Vietnam	22–35+	Unclear	24–32 weeks	ADA IADPSG	Universal	6.1 20.3	Not reported	Not reported	
22. Baci et al. (2013)	Turkey	18–45	Not reported	24–28 weeks	ADA	Universal	1.95	Not reported	Not reported	
23. Karcaaltincaba et al. (2009)	Turkey	14–49	Not reported	Unclear	ADA NDDG	Universal	4.48 3.17	Not reported	Not reported	
24. Kosus et al. (2012)	Turkey	Unclear	Unclear	24–28 weeks	ADA NDDG	Universal	8.1 5.6	Not reported	Not reported	
25. Erem et al. (2002)	Turkey	<20–30+	Unclear	24–32 weeks	NDDG	Universal	1.23	Not reported	Not reported	
26. Tanir et al. (2005)	Turkey	27.3–37.9	Unclear	Unclear	ADA	Universal	3.10	Not reported	Not reported	
27. Hadaegh et al. (2005)	Iran	19–30	Not reported	Unclear	ADA NDDG	Universal	8.90 6.30	Not reported	Not reported	
28. Hossein-Nezhad et al. (2006)	Iran	15–45	Unclear	Unclear	ADA NDDG	Universal	4.70 3.97	Not reported	Not reported	
29. Keshavarz et al. (2005)	Iran	20–35	Unclear	13.4–28.6 weeks	ADA	Universal	4.80	Not reported	Not reported	
30. Ranchod et al. (1991)	South Africa	Unclear	Not reported	Unclear	EASD WHO	Universal	1.56 3.78	0.23%	Not reported	
31. Anzaku and Musa (2013)	Nigeria	21–40	Unclear	24–28 weeks	WHO	Universal	1.60	Not reported	Not reported	
32. Olarinoye et al. (2004)	Nigeria	18–41	Mean: 1.3	Unclear	WHO NDDG	Universal	6.45 2.02	Not reported	Not reported	
33. Ozumba et al. (2004)	Nigeria	15–54	0 to 4=81% >4=19%	Unclear	WHO	Universal	1.01	0.65%	Not reported	
34. Balaji et al. (2012)	India	Mean 23.8 (±3.48)	Not reported	24–28 weeks	WHO	Universal	10.5	Not reported	Not reported	
35. Seshiah et al. (2008), (2009)	India	19–27	Not reported	16.9–34.3 weeks	WHO	Universal	13.90	Not reported	Not reported	
36. Zargar et al. (2004)	India	18–38	Mean: 2.1	~24 weeks	ADA WHO	Universal	3.10 4.40	Not reported	Not reported	
37. Dahanayaka et al. (2012)	Sri Lanka	19–≥35	1=42.2%2 to 4=55.8%>4=2.0%	24–28 weeks	WHO IADPSG	Universal	7.16 8.89	Not reported	Not reported	
38. Sayeed et al. (2005)	Bangladesh	18–44	Not reported	<26 & >26 weeks	WHO	Universal	8.20	Not reported	Not reported	
39. Yang et al. (2009)	China	20–>30	Not reported	Unclear	ADA	Universal	4.35	Not reported	Not reported	Population-
40. Yang et al. (2002)	China	26–28	Not reported	26–30 weeks	WHO	Universal	1.84	Not reported	Not reported	based studies
41. Zhang et al. (2011)	China	<25–>35	Not reported	26–30 weeks	WHO	Universal	4.90	Not reported	Not reported	
42. Seyoum et al. (1999)	Ethiopia	20–35	Unclear	Unclear	WHO	Universal	3.70	Not reported	Not reported	
43. McCarthy et al. (2010)	Argentina	13–45	Unclear	24–28 weeks	WHO	Universal	5.80	Not reported	Not reported	
44. Schmidt et al. (2000)	Brazil	22–33	Unclear	21–28 weeks	WHO	Universal	0.40	Not reported	Not reported	
45. Davilla et al. (2011)	Cuba	Unclear	Null=14% >1=86%	<20 to>32 weeks	WHO modified	Universal	17.25	0.70%	Not reported	

T1DM, type 1 diabetes mellitus; T2DM, type 2 diabetes mellitus; GDM, gestational diabetes mellitus; DM, diabetes mellitus; WHO, World Health Organization; ADA, American Diabetes Association, ADIPS, Australian Diabetes in Pregnancy Society; NDDG, National Diabetes Data Group; DIPSI, Diabetes in Pregnancy Study Group India; EASD, European Association for the Study of Diabetes; IADPSG, International Association of Diabetes and Pregnancy Study Groups.

### Risk of bias

The studies included were assessed for risk of bias by two reviewers independently (LK, NB). The validity of methodology, its appropriateness and reporting of results were assessed (20, 21). Seven criteria were used to assess three risks of biases, namely measurement bias, selection bias and attrition bias.

## Results

**Fig. 1:**
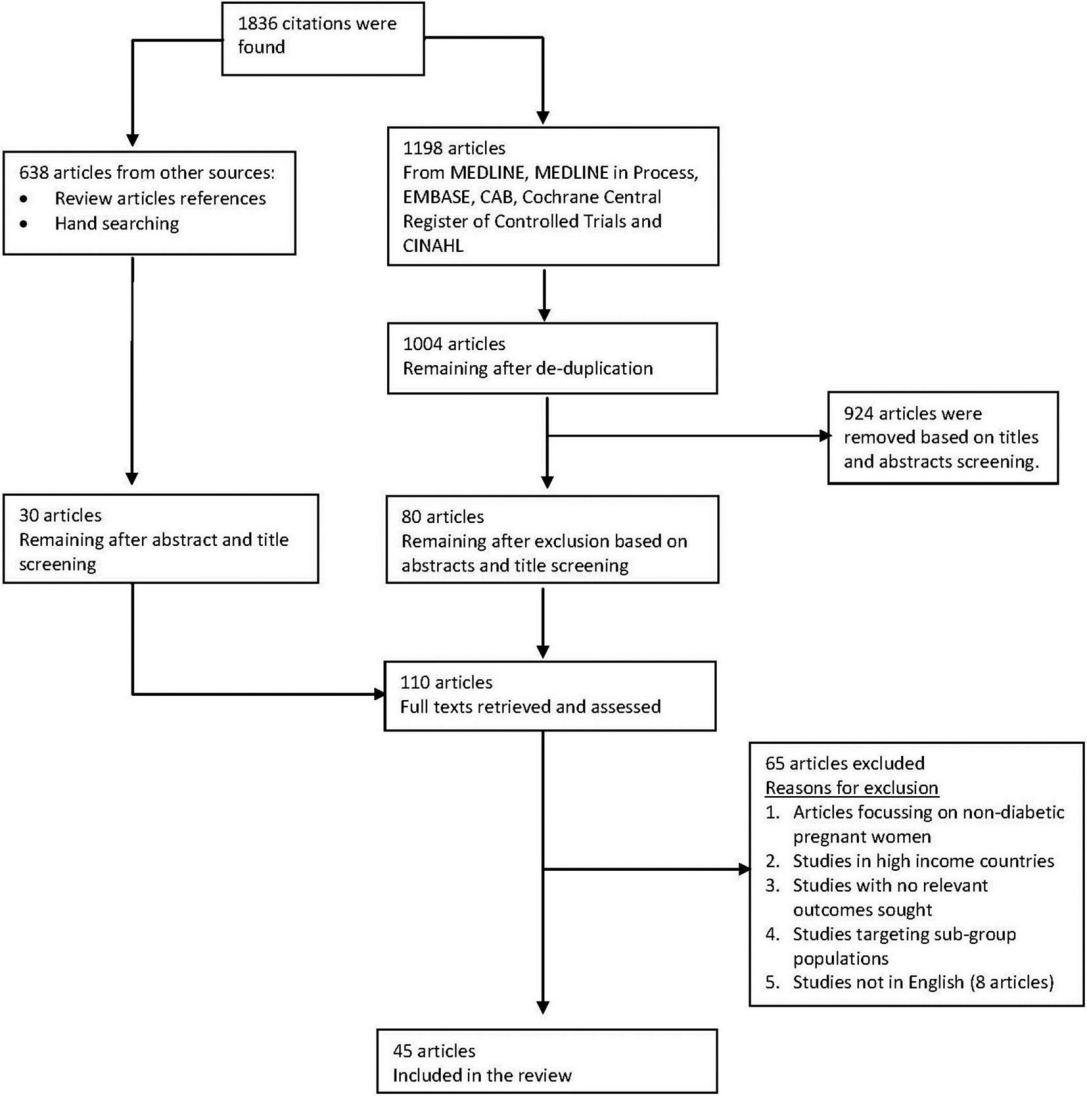
Flow chart of study selection. In general, studies were excluded based on participants (if it included women who were not pregnant or those beyond 1 year in the postpartum period), study design (if these were commentaries, letters of correspondence, systematic reviews), outcome measure (if it did not include relevant outcomes sought) and was not a low- and middle-income country as defined by the World Bank.

The searches conducted yielded 1,836 citations. After screening titles, abstracts and full texts, 45 studies (Fig. 1) with 281,661 participants were included. Among the excluded studies were eight non-English articles, with two each in German and French, and one each in Norwegian, Spanish, Persian and Portuguese after abstract and title screening.

### Characteristics of included studies

**Fig. 2:**
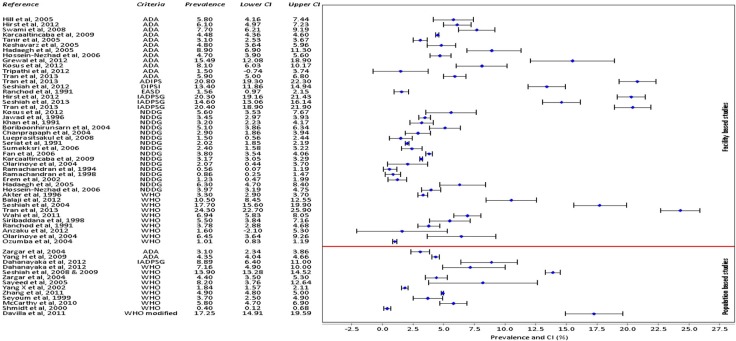
DGM prevalence and confidence intervals (CI).

The included studies were from Pakistan (22–24), India (25–37), Sri Lanka (38, 39), Bangladesh (40), Thailand (41–45), China (46–49), Vietnam (50, 51), Turkey (52–56), Iran (57–59), South Africa (60), Ethiopia (61), Nigeria (62–64), Argentina (65), Brazil (66) and Cuba (67) (Table 1). The largest number of studies (12) came from India. The studies included were either cohort or cross-sectional studies. About 60% of the studies (26 studies) were based in urban areas. Two studies from Bangladesh and Ethiopia specified a rural population base. Three other studies reported that both urban and rural areas were covered, while in 14 studies, the setting was not described. In Fig. 2, the included studies are grouped into 33 studies which were facility-based and 12 which were population-based (also seen Table 1). Facility-based studies were considered as those sampling one or a few hospitals/clinics, and were not reported to be representative of the total targeted population in the study district/region. Population-based studies included those that reported to have sampled the whole population of interest in the selected district(s)/region or those which reported systematic sampling of the population in a region/district likely to be representative of the total targeted population (Table 1, sampling frame). Sample sizes across all the studies included varied from as low as 172 to 105,472 participants.

Table 2 shows outcome measures of interest across the various studies. In general, studies varied from reporting on prevalence only to reporting on prevalence, risk factors, pregnancy outcomes and interventions (data not shown). Fifteen studies reported on prevalence only (24, 25, 28–31, 34, 35, 37, 49, 53, 60, 61, 66, 67), 10 studies on prevalence and risk factors only (38, 40, 41, 46, 47, 50, 52, 54, 57, 62), 12 studies on prevalence, risk factors and pregnancy outcomes/obstetric complications (21, 25, 26, 35, 41–44, 54, 57, 62–64), and eight studies on prevalence, pregnancy outcomes/complications and some form of intervention (22, 31, 32, 38, 47, 50, 58, 59). The interventions in the latter group included diet/medical nutrition therapy only, insulin only or combined diet and insulin therapy.

Maternal age ranged from as low as 13 years in Argentina to 54 years of age in Nigeria (Table 2). Parity was poorly reported by only 10 studies. Among these, GDM prevalence was higher in women who had given birth to one child or more, than in those giving birth for the first time (Table 2). Gestational age at diagnosis of GDM was only reported by about 60% of the studies included. The majority of these studies reported on a diagnosis being made between the 24th and 32nd gestational weeks.

The screening criteria used were reported by all the studies included. Thirty-nine studies used universal screening of participants while the remaining six studies used selective screening of pregnant women at high risk of GDM (Table 2), except those with multiple pregnancies and other predisposing medical conditions. The most common diagnosing criteria used were the WHO criteria, followed by the NDDG criteria, and then the ADA criteria. The IADPSG, ADIPS, DIPSI, EASD and modified WHO criteria were less popular diagnosing criteria. GDM was the most frequently documented type of diabetes in pregnancy reported by all the studies, while the prevalence of pre-existing type 1 or type 2 DM was only reported in seven studies.

### Prevalence

There were 58 observations of GDM prevalence from the 45 studies, because more than one criterion was used by some studies (Table 2). Prevalence using the ADA criteria (15 observations) ranged from 1.50 to 15.50%. With the NDDG criteria (16 observations), prevalence ranged from 0.56 to 6.30%, the WHO criteria (19 observations) ranged from 0.4 to 24.30%, and the IADPSG criteria (four observations) ranged from 8.9 to 20.4%. EASD, ADIPS, DIPSI and WHO modified criteria each had only one observation with prevalence of 1.56, 20.8, 13.4 and 17.25% reported, respectively. GDM prevalence rates and their confidence intervals are summarised in Fig. 2.

Prevalence of type 1 and 2 DM were reported as ranging from 0.20 to 0.70%. Neither postpartum DM after 6 weeks nor maternal mortality due to any type of DM was reported.

**Fig. 3:**
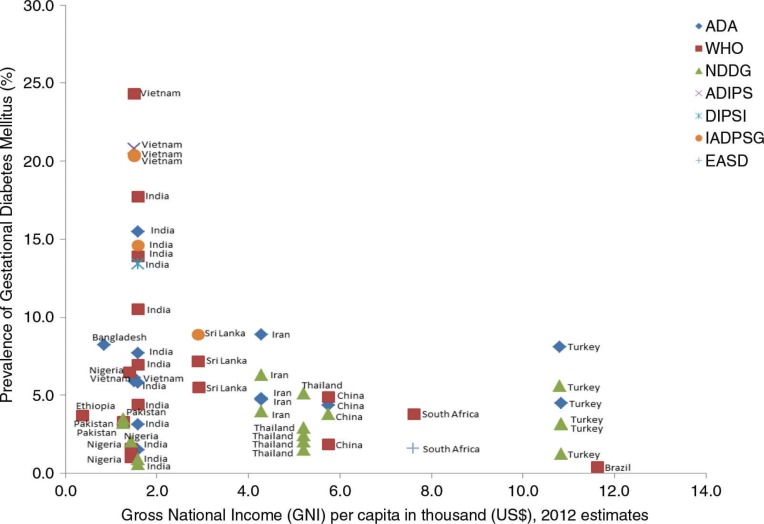
Prevalence of gestational diabetes mellitus against gross national income per capita in thousands (US$).

The association between GDM and GNI per capita is shown in Fig. 3. A significant negative correlation is seen (*B*=−0.611; *R*=0.358; *p*=0.007). Three studies (28, 50, 51) are clear outliers on the graph, and without these the suggestion of an association is further reduced (*B*=−0.314; *R*=0.291; *p*=0.042).

### Risk of bias

**Fig. 4:**
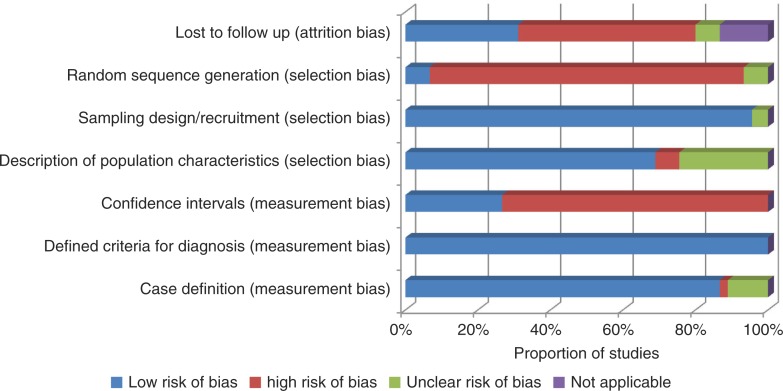
Risk of bias summary (bias considerations vs. proportion of studies).

A summary of the risk of bias in included studies is shown in Fig. 4. The diagnostic criteria used were well defined in all of the studies. A total of 86% (39 studies) had clear case definitions, and 95% (43 studies) reported clearly on the sampling design and recruitment processes used. However, studies were subject to a high risk of bias in a few parameters. Confidence intervals were only reported by 26% (12 studies). Only 7% (three studies) randomly sequenced the selection of participants and 31% (14 studies) reported on loss to follow-up.

## Discussion

Our review is the first to systematically summarise the published literature on prevalence of GDM and type 1 and 2 DM in pregnancy in low- and middle-income countries. We found 45 studies recording the prevalence of DM in pregnancy which passed our selection criteria. They only cover a select number of countries and large areas of Africa and Asia are not covered by existing studies. GDM prevalence ranged from 0.40 to 24.3% and pre-existing DM (type 1 and 2) ranged from 0 to 0.7%.

It is well known that a wide variation of DM is seen across countries (68). High prevalence rates are reported to occur among Asian, Latin America and Middle Eastern populations. Ethnicity (69) and geographical variation (70, 71) are important factors and are well documented across high-income countries such as Bahrain (13.5%) (72), Qatar (16.3%) (73), United Arab Emirates (14.2–23.1%) (74), Hong Kong (14.2%) (75), Ireland (9.4–12.4%) (76), Israel (6.07%) (77) and the United States (2–10%) (78). In our study, the highest prevalence of GDM was reported from Vietnam (50, 51), India (28–30, 34, 35, 37) and Cuba (67), followed closely by Bangladesh (40) and Iran (57). Although the prevalence ranges that we found were wide, most were fairly consistent with the global GDM prevalence ranging between 1 and 14% (70), except those from Vietnam which were unusually high. Although data is sparse, we also found one unexpectedly high level of GDM prevalence reported in Nigeria of 6.45%, which is comparable to the levels found in some of the Asian countries with high ethnic predisposition to DM. The comparative prevalence of DM in Africa's general population is 5.7% (19.8 million adults), which is the lowest in all the regions, and also lower than the average global prevalence estimated at 8.3% (79). Nonetheless, this finding from the study from Nigeria was not corroborated by using different diagnostic criteria (Table 2), and a comparison was not done on the same population. The study was also facility-based and participants had been randomly allocated to either WHO (75 g OGTT) or NDDG (100 g OGTT) study arms for comparison (63). In general, facility-based studies have higher GDM prevalence levels than population-based studies due to increased likelihood of patients presenting at health facilities. Hence, caution should be taken in the interpretation of this result.

One of the major limitations our review highlights is the difficulty in determining prevalence due to the lack of consistency in the use of diagnosing criteria (e.g. ADA, WHO, NDDG, IADPSG). The various diagnostic criteria use different loading doses for OGTT and different thresholds for fasting times (between 1 and 3 hours). The number of abnormal plasma glucose values considered to be adequate for a diagnosis also change when using the various criteria. For example, the ADA criteria uses a loading dose of 100 g for OGTT, and allows for two or more abnormal values for a diagnosis to be made, whereas the WHO criteria recommends 75 g for OGTT allowing only one or more abnormal values to be used for diagnosis (13, 80). This is compounded by the differences in sensitivity and specificity between the diagnosing criteria. In an ideal setting, a clinical test with the highest accuracy to identify patients with a disease (sensitivity) and those without a disease (specificity) is usually desirable. However, most clinical tests do not always satisfy this ideal. A systematic review by Donovan et al. investigated the sensitivity and specificity of the various tests for diagnosing GDM using different thresholds (81). The authors found that at the threshold of 7.8 mmol/L, the ADA criteria had the highest sensitivity of 88 (86–97)%, followed by NDDG criteria at 85 (73–92)% then the WHO criteria at 70 (43–85)%. IADPSG had very low sensitivity at 12 (7–18)%. Conversely, the IADPSG had the highest specificity at 97 (95–98)%, compared to that of the WHO, ADA and NDDG criteria at 89 (73–94)%, 84 (79–87)% and 83 (78–87)%, respectively. To date, there is still a lack of clarity as to which diagnostic criteria should be used. The various debates are centred around frequency of diagnosis by different criteria, cost effectiveness and the dilemma of undiagnosed cases who are at risk of poor maternal and perinatal outcomes if they remain undetected, and where care may be sub-optimal (82, 83).

We considered the possibility of conducting meta-analyses in this review but did not do these as there is expected to be considerable variability across ethnic groups and countries. Pooling of data even from a single country was not done due to the variation in use of diagnostic criteria and lack of a control group for most studies.

Pre-existing (type 1 and 2) DM in pregnancy was reported by very few studies. Since the highest burden of type 2 DM exists in low- and middle-income countries also affects women of reproductive age (2), it is surprising that studies did not capture these women while pregnant. A number of the studies in our review did however specifically exclude women with pre-existing diabetes. No studies reported on postpartum type 2 diabetes (after 6 weeks) indicating an acute lack of information on women followed up to 1 year after childbirth. This could be linked to poor postnatal attendance and resource constraints in health services (84, 85). There were no studies reporting on maternal deaths due to diabetes. These could be due to several reasons such as deaths being masked by misclassification or inappropriate coding or missed because they were late maternal deaths that occurred within 1 year after delivery. Undiagnosed diabetes may also be another contributing factor. Up to 50% of people living with diabetes worldwide are currently undiagnosed (1), and it is known that diabetes can lead to complications in pregnancy such as pregnancy-induced hypertension, pre-eclampsia, postpartum haemorrhage and increased risk of infections. It is possible that in undiagnosed diabetic patients who develop complications in pregnancy and succumb to it, these complications could have been attributed as the main cause of death and not diabetes which was the underlying cause of death.

A statistically significant inverse relationship was seen between the prevalence of GDM and a country's wealth as measured by the GNI. It was expected that with increasing wealth GDM would become more prevalent, and/or the functionality of the health system would improve, thus increasing the number of cases picked up through screening. Although conclusions from this rather crude analysis cannot be drawn, the trends observed would be worth investigating further to understand and plan for future health needs in countries with transitioning economies.

A number of limitations are inherent in our review design. We were unable to assess papers in languages other than the English language. Of the eight full-text papers excluded for this reason, these included papers in German (2), French (2), Norwegian (1), Spanish (1), Persian (1), and Portuguese (1) languages. The likelihood is that studies from Latin America, French-speaking African countries and the Middle East are most likely to be underrepresented if published in regional/local non-English journals. We did not restrict the dates for our search, and the majority of the studies were from the last 15 years. A few studies dated to the early 1990s and the observed demographic changes in tendency to develop DM in young adults could have affected any patterns we might have observed. Studies included in this review may have been subjected to some bias, primarily in the form of selection bias. These were in terms of how participants were selected (Fig. 4) and whether they were representative of the target population as a whole which may have affected the estimates of prevalence obtained. Commonly, selection bias occurs when the participants studied are not representative of the target population about which conclusions are to be drawn. For example, if an investigator wishes to estimate the prevalence of disease X in adult residents of a certain town/city/region, s/he may attempt to do this by selecting a random sample from all the adults enrolled with several local health facilities, and then recruit them. However, this design, which is used in some studies included in this review, would be systematically excluding participants who do not access health facilities and therefore impact on the results obtained. Eliminating selection bias in epidemiological studies is therefore critical for accurate results and should always be considered when defining a study sample. In addition, including confidence intervals which describe a range within which one can reasonably expect the true value to lie would be important. Unfortunately, this was not documented in a number of studies included in this review.

## Conclusion

DM is a growing public health problem in low- and middle-income nations. This systematic review has highlighted the disparate and piecemeal data available from the published literature on prevalence of DM during pregnancy in these countries. Without such data, it will be difficult to make rational decisions for allocating precious funding within expanding health systems. A global consensus on the diagnostic criteria for DM is urgently required so that the public health burden of the condition can be assessed. Studies of prevalence should capture populations beyond those presenting in health facilities, as little is known about undetected DM in pregnancy. The current focus on GDM needs to be extended to also capture diabetes in women of reproductive age especially just before pregnancy and in the months after delivery, as these are the times when interventions can optimise the health of women and maximise the likelihood of a healthy foetus.
